# The τCstF-64 Polyadenylation Protein Controls Genome Expression in Testis

**DOI:** 10.1371/journal.pone.0048373

**Published:** 2012-10-26

**Authors:** Wencheng Li, Hsiang-Jui Yeh, Ganesh S. Shankarling, Zhe Ji, Bin Tian, Clinton C. MacDonald

**Affiliations:** 1 Department of Biochemistry and Molecular Biology, UMDNJ-New Jersey Medical School, Newark, New Jersey, United States of America; 2 Department of Cell Biology & Biochemistry, Texas Tech University Health Sciences Center, Lubbock, Texas, United States of America; Ecole Normale Superieure de Lyon, France

## Abstract

The τCstF-64 polyadenylation protein (gene symbol *Cstf2t*) is a testis-expressed orthologue of CstF-64. Mice in which *Cstf2t* was knocked out had a phenotype that was only detected in meiotic and postmeiotic male germ cells, giving us the opportunity to examine CstF-64 function in an isolated developmental system. We performed massively parallel clonally amplified sequencing of cDNAs from testes of wild type and *Cstf2t^−/−^* mice. These results revealed that loss of τCstF-64 resulted in large-scale changes in patterns of genome expression. We determined that there was a significant overrepresentation of RNAs from introns and intergenic regions in testes of *Cstf2t^−/−^* mice, and a concomitant use of more distal polyadenylation sites. We observed this effect particularly in intronless small genes, many of which are expressed retroposons that likely co-evolved with τCstF-64. Finally, we observed overexpression of long interspersed nuclear element (LINE) sequences in *Cstf2t^−/−^* testes. These results suggest that τCstF-64 plays a role in 3′ end determination and transcription termination for a large range of germ cell-expressed genes.

## Introduction

Polyadenylation is the co-transcriptional process by which most mRNAs form their 3′ ends in eukaryotic organisms. The polyadenylation machinery is highly conserved in all eukaryotes [1], highlighting the fundamental importance of polyadenylation for gene expression and cell viability. Further, mRNA processing in general and polyadenylation in particular are forces that modify chromatin structure, changing the dynamics of gene expression [2], [3], [4], [5]. Together, these functions imply that polyadenylation is necessary for global gene expression, chromatin function, and genomic integrity.

At least five multi-protein factors and up to 85 proteins are involved in polyadenylation [6], [7]. Of these factors, the cleavage specificity factor (CstF) appears to have key functions in regulating alternative polyadenylation [5], in coupling polyadenylation to transcription termination [8], and in integrating polyadenylation with DNA damage responses [9]. CstF is composed of three polypeptides, of which the 64,000 *M_r_* polypeptide, CstF-64 (gene symbol *CSTF2*) is the RNA-binding component of CstF that binds to the GU-rich downstream polyadenylation element [10], [11], [12], [13], resulting in changes in polyadenylation site choice [14], [15].

Mammals have two paralogous genes encoding CstF-64: *CSTF2* is X-linked and encodes the somatic CstF-64 that is expressed in all tissues [16]. In contrast, *CSTF2T* (*Cstf2t* in mice) is an autosomal retrotransposed gene that encodes τCstF-64 [17], [18]. τCstF-64 is expressed at highest levels in testis and brain, and at lower levels in other tissues and cell types [7], [19]. In testis, τCstF-64 is expressed exclusively in male germ cells, because male sex chromosome inactivation (MSCI) results in transcriptional inactivation of the somatic CstF-64 [20].

Congruent with this hypothesis, although τCstF-64 is expressed in multiple tissues, targeted deletion of *Cstf2t* revealed that the function of τCstF-64 was primarily in spermatogenesis [21]. Male mice homozygous for *Cstf2t^tm1Ccma^ (i. e., Cstf2t^−/−^* mice) exhibited male infertility and spermatogenic defects resembling human oligoasthenoteratozoospermia *(ibid.)*. While spermatogenesis was greatly disrupted, a few morphologically defective but active spermatozoa were produced [22], [23]. This suggested that deletion of τCstF-64 resulted in systematic alterations in gene expression, but not absolute loss of expression of key spermatogenic genes. Therefore, we wanted to examine changes in global gene expression that correlated with the absence of τCstF-64 polyadenylation function.

**Figure 1:**
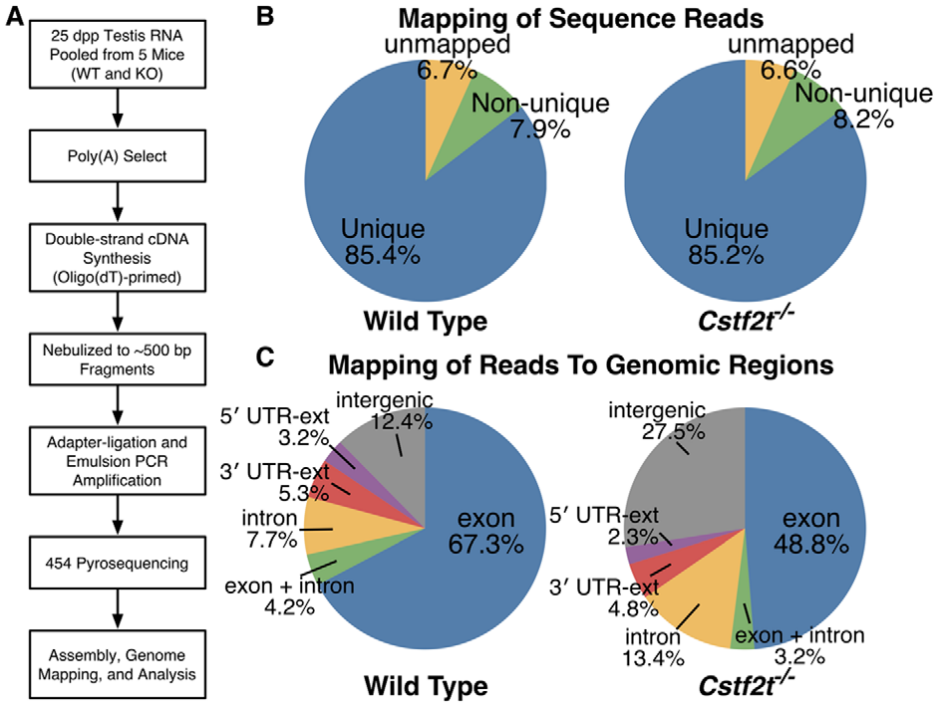
High-throughput cDNA sequencing (RNA-seq) finds significant differences between wild type and *Cstf2t^−/−^* mouse testis RNAs. (**A**) RNA was pooled from testes of five 25 dpp mice of either wild type or *Cstf2t^−/−^* genotype, cDNA synthesized, and high-throughput sequencing performed (see Materials and Methods). (**B**) RNA-seq from wild type (∼55,000 reads) and *Cstf2t^−/−^* (∼77,000 reads) mouse testis samples were not biased when mapped to the mouse genome. 454 sequencing reads were mapped to the mouse genome (Mouse Genome Assembly version mm8) using BLAT [24]. Pie graphs show that similar proportions of reads mapped to either unique genomic regions (blue), multiple regions (non-unique, green), or could not be mapped to known regions (unmapped, tan) in samples from wild type or *Cstf2t^−/−^* mouse testes. The proportion of uniquely mapped reads has no statistical difference between wild type and Cstf2t−/− mice (85.4% vs. 85.2%; P = 0.14, Fisher's exact test). (**C**) Introns and intergenic regions were more highly expressed in testes of *Cstf2t^−/−^* mice, while exons were less expressed. Pie graphs show percentages of reads that were uniquely mapped to different regions of the genome for wild type and *Cstf2t^−/−^* mice. Exon (blue), reads fully aligned to exons; exon & intron (green), reads aligned to both exonic and intronic regions; intron (tan), reads fully aligned to introns; 3′ UTR-ext (orange) and 5′ UTR-ext (purple), reads aligned to within 4 kb downstream of 3′ UTR or 1 kb upstream of the 5′ UTR, respectively; intergenic (grey), reads aligned to regions not within annotated genes or their extended regions. The difference of proportion of reads mapped to different genomic regions is significant: P <10–323 for both the intergenic region and intronic region (Fisher's exact test, exon region used as control).

Here we compare results of massively parallel clonally amplified sequencing of cDNAs from testes of wild type and *Cstf2t^−/−^* mice. We found that poly(A)-selected cDNAs from *Cstf2t^−/−^* mouse testis contained significantly less representation from annotated exonic regions, but more representation from intronic and intergenic regions. In agreement with these data, we observed down-regulation of intronless small genes (ISGs) in *Cstf2t^−/−^* mouse testis, and concomitant increase in the amount of read-through transcription, suggesting these effects were due to aberrant transcriptional termination. Finally, we observed increased representation of long interspersed nuclear elements (LINEs) L1 in *Cstf2t^−/−^* mouse testis, but not of other repetitive elements such as short interspersed nuclear elements (SINEs) such as B2 and B4 elements or LTR elements. This suggests that τCstF-64 represses L1 elements selectively in mouse testes. Together, these data support a model in which τCstF-64 in male germ cells – and, by extension, the process of polyadenylation in all cells – is critical for global control of genomic gene expression.

**Figure 2:**
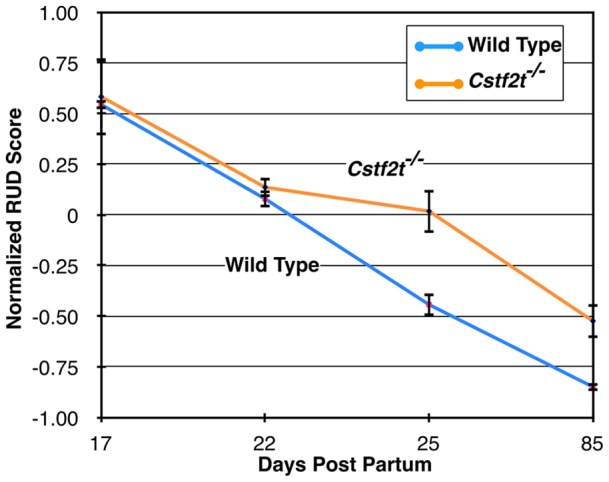
Relative usage of distal poly(A) sites (RUD) decreases throughout testis development, but less so in *Cstf2t^−/−^* mouse testes. The Y-axis is the mean RUD score which reflects relative usage of distal poly (A) sites [26], [27]. RUD values were based on three replicates for the 17, 22 and 25 dpp time points and two replicates for the 85 dpp time point. Error bars indicate the standard deviation (P values for T-test are 0.69, 0.32, 0.006 and 0.15 for 17, 22, 25 and 85 dpp, respectively comparing KO and WT). A step-wise decrease can be seen, indicating progressive shortening of 3′ UTRs or more usage of proximal poly(A) sites from 17 to 85 days postpartum (dpp). However, *Cstf2t^−/−^* differs from wild type starting at 25 dpp through 85 dpp.

## Materials and Methods

### Animals

Animal studies were performed in accordance with protocols according to National Institutes of Health guidelines and approved by the Texas Tech University Health Sciences Center Institutional Animal Care and Use Committee. The *Cstf2t^tm1Ccma^* mice used in these studies were of mixed C57BL/6–129SvEv background. All genotyping was done as described previously [21].

**Figure 3:**
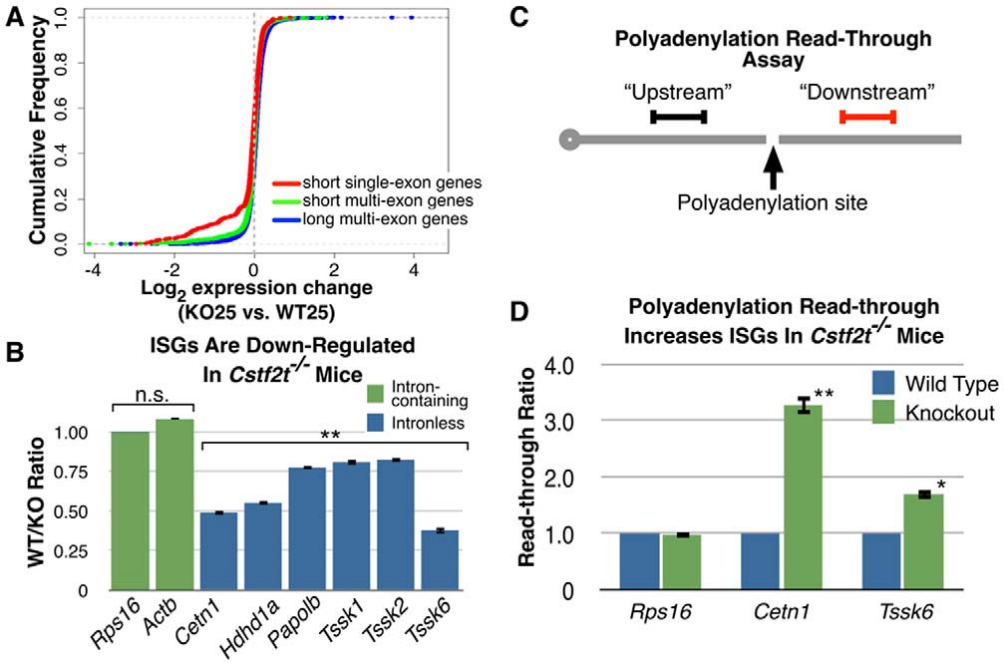
ISGs are down-regulated and have increased read-through in *Cstf2t^−/−^* mouse testes. (**A**) Cumulative frequency of microarray log_2_ mRNA expression changes of *Cstf2^−/−^* (KO25) versus wild type (WT25) mouse testis at 25 dpp. Short genes were defined as the lowest 20% in length, with a cutoff of 6658 bp or shorter. Indicated are long multi-exon genes (11,451 genes, blue), short multi-exon genes (2,324 genes, green), and short single-exon genes (541 genes, red). There are 276 short single-exon genes in the region between -2 and 0 log_2_ expression change. P values are 4.2×10^−4^ between short single-exon and short multi-exon genes and 1.0×10^−15^ between long multi-exon and short-multi-exon genes by a K-S test. (**B**) qRT-PCR was performed using primers specific for the indicated genes (see Table S1) normalized to *Rps16*. Each bar represents the amount (in percent) of the indicated mRNA in 25 dpp *Cstf2t^−/−^* mouse testis RNA compared to wild type. The asterisks indicate values that are significantly different (P<0.001) from Rsp16 and Actb by ANOVA (Bonferroni multiple comparisons test). (**C**) Polyadenylation read-through assay. Random-primed cDNA is made from RNA from wild type or *Cstf2t^−/−^* mouse testes. qRT-PCR is then performed using primer pairs within the body of the gene (“Upstream”) or downstream of the polyadenylation site (“Downstream”). An increase in read-through is measured as in increase in the downstream value compared to the upstream value in *Cstf2t^−/−^* mice after normalization. (**D**) Read-through increases for ISGs in *Cstf2t^−/−^* mouse testes. The polyadenylation read-through assay described in (C) was performed on the indicated genes and normalized to 1.0 in the wild type mice. The asterisk (P<0.05) and double asterisk (P<0.01) indicate values that differ significantly from the wild type by a Student's t-test.

### RNA Preparation and Sequencing

Total RNA was extracted from the testes of 25 day postpartum (dpp) wild type or *Cstf2t^tm1Ccma/tm1Ccma^ (Cstf2t^−/−^)* mice using the TRIzol reagent (Invitrogen, Carlsbad, CA), treated with DNase (Ambion, Austin, TX), poly(A)^+^ RNA prepared using oligo(dT) columns (New England Biolabs, Ipswich, MA), and oligo(dT)-primed double-stranded cDNA synthesized using the Just cDNA Double-Stranded cDNA Synthesis Kit (Agilent Technologies, Santa Clara, CA, Fig. 1A). Resulting cDNA (4 µg) was nebulized to produce fragments of ∼500 bp, adapters were ligated onto the cDNA fragments, and emulsion-PCR performed to amplify the cDNA products. Massively parallel pyrosequencing was performed using the 454 Titanium protocol (454 Life Sciences, Branford, CT).

**Figure 4:**
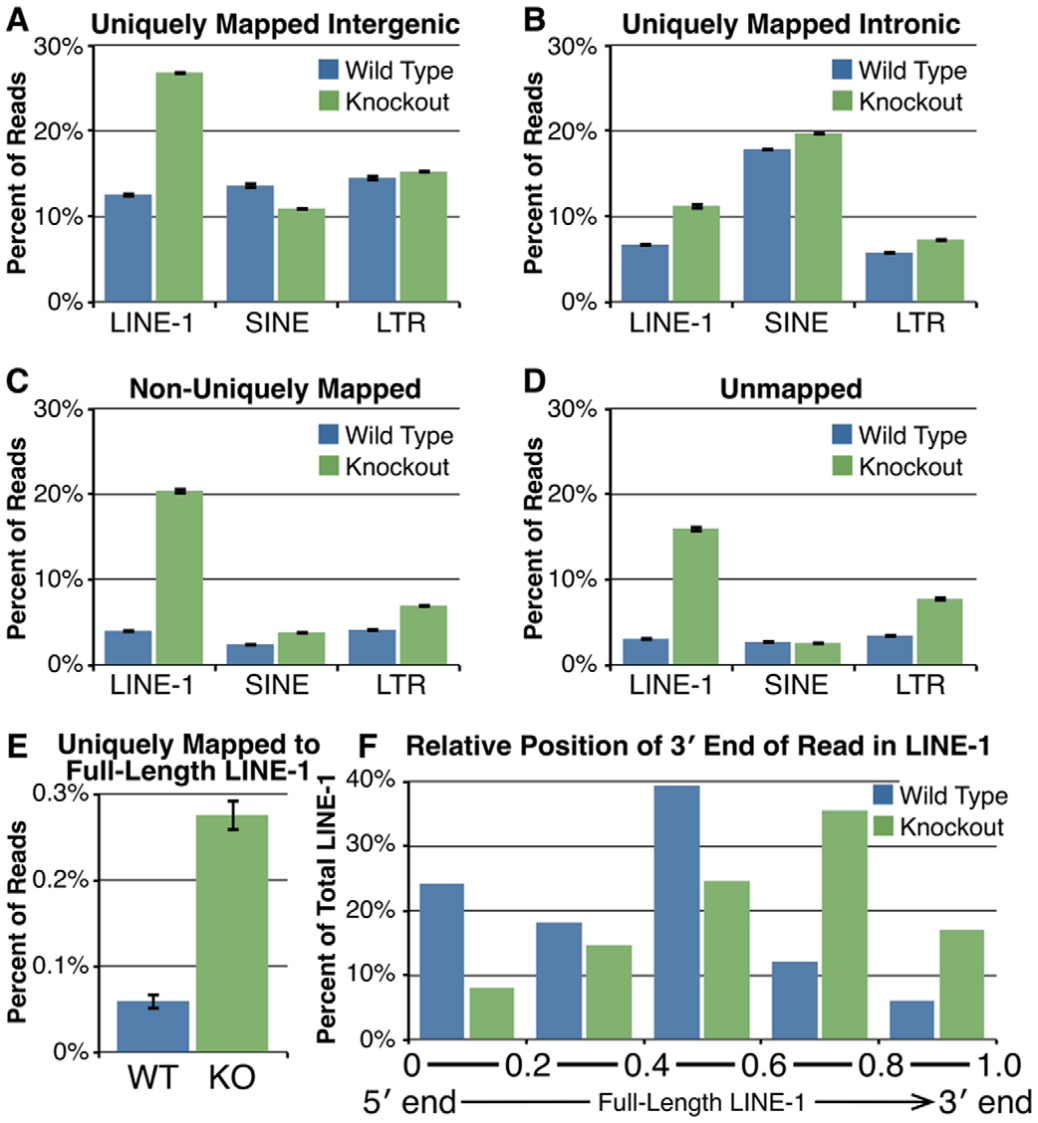
LINE-1 mRNAs are over-represented in *Cstf2t^−/−^* mouse testes. (**A**) Comparison of reads uniquely mapped to different transposable elements in intergenic regions in 25 dpp wild type (blue bars) and *Cstf2t^−/−^* (green bars) mice. Error bars denote standard deviations. LINE-1, SINE and LTR classes of transposable elements were identified using RepeatMasker [46]. LINE-1 mRNAs are 2.1-fold more abundant in *Cstf2t^−/−^* mice than in wild type (P = 9.0×10^−141^ by Fisher's exact test), while SINEs are only 0.8-fold different (P = 4.4×10^−9^) and LTRs are 1.05-fold different (P = 0.15). (**B**) Comparison of reads uniquely mapped to different transposable elements in intronic regions in wild type and *Cstf2t^−/−^* mice. LINE-1 mRNAs are 1.7-fold more abundant in *Cstf2t^−/−^* mice than in wild type (P = 5.3×10^−26^), SINEs are 1.1-fold different (P = 1.6×10^−3^) and LTRs are 1.3-fold different (P = 4.8×10^−5^). (**C**) Comparison of reads mapped to different transposable elements in multiple genomic regions in wild type and *Cstf2t^−/−^* mice. LINE-1 mRNAs are 5.1-fold more abundant in *Cstf2t^−/−^* mice than in wild type (P = 1.1×10^−173^), SINEs are 1.6-fold different (P = 1.2×10^−5^) and LTRs are 1.7-fold different (P = 1.3×10^−11^). (**D**) Comparison of unmapped reads that align with transposable elements in wild type and *Cstf2t^−/−^* mice. LINE-1 mRNAs are 5.2-fold more abundant in *Cstf2t^−/−^* mice than in wild type (P = 1.1×10^−112^), SINEs are 1.0-fold different (P = 0.76) and LTRs are 2.2-fold different (P = 8.2×10^−21^). (**E**) Percent of uniquely mapped reads that partially or completely mapped to full length (≥6 kb) LINE-1 sequences in the genome in 25-dpp wild type (blue bars) or *Cstf2t^−/−^* (green bars) mouse testes. LINE-1 mRNAs are 4.6-fold more abundant in *Cstf2t^−/−^* mice than in wild type (P = 7.8×10^−22^, Fisher's exact text). Error bars denote standard deviations of proportion using the formula √[p*(1-p)/N] where p is the proportion of reads mapped to one type of repeat sequence and N is the total number of reads used for mapping. (**F**) Location of the 3′ end of the sequence reads in along uniquely-mapped full length LINE-1 sequences (from b). Each LINE-1 sequence was evenly divided into 5 regions from 5′ to 3′, and the number of reads whose 3′ ends mapped to each region was determined. The distribution of 3′ ends of reads of WT was significantly different than that of KO (P = 0.002, Chi-squared test).

### Sequence Analyses

454 sequencing reads were mapped to the mouse genome (Mouse Genome Assembly version mm8) using BLAT [24]. For reads with multiple hits, an alignment score was calculated for each hit which was based on the difference of the number of mismatched nucleotides from the number of matched nucleotides. If the alignment score of the best hit was greater than that of the second best hit by ≥10, the read was considered as uniquely mapped to the genome. Uniquely mapped reads were annotated by the gene structure based on RefSeq sequences. Identification of transposable element sequences was based on RepeatMasker [25]. Uniquely mapped reads were also compared to the LINE-1 sequences in the genome based on the LINE track in UCSC genome browser. Reads mapped to the LINE-1 sequences with ≥6 kb were used to support expression of full-length LINE-1 and the 3′ end positions of the reads along the LINE-1 sequences were further examined. Data will be deposited into the NCBI Sequence Read Archive (SRA), and a BED format file will be available upon request.

### Relative Usage of Distal Poly(A) Sites

Relative usage of distal poly(A) site (RUD) scores were determined from our previous microarray data [21] for 17, 22, 25, and 85 day post partum (dpp) mouse testis RNA. Each gene was assigned a RUD score value that reflected the relative 3′ untranslated region (UTR) length in each sample [26], [27]. The mean RUD of all genes in a sample is the RUD for the entire sample. To decrease sample bias, RUD scores were normalized to genes that do not exhibit alternative polyadenylation as described *(op. cit.).*

### Quantitative RT-PCR (qRT-PCR)

Complementary DNA was prepared from wild type or *Cstf2t^−/−^* testes as described [21]. Experiments were performed using RNA from testes of at least three wild type and three Cstf2t−/− mice at 25 dpp. Real time PCR was performed using the indicated primers (Table S1) with a 96-well format ABI 7500 Real-Time PCR System (Applied Biosystems, Foster City, CA), with iTaq SYBR Green Supermix with ROX (Bio-Rad, Hercules, CA). PCR conditions were 95°C for 3 min, followed by 40 cycles of 95°C (15 sec) and 55°C (45 sec), followed by a dissociation stage. C_t_ data were normalized to the ribosomal protein *Rps16* mRNA, which was run in every experiment.

## Results and Discussion

### High-throughput RNA Sequencing Reveals That Intergenic Regions and Introns Are Overrepresented in Testes of *Cstf2t^−/−^* Mice

Many aspects of mRNA polyadenylation are different in mammalian male germ cells from somatic cells. In germ cells, RNA signals differ [28], alternative sites are used [26], [27], [29], and germ cell variants of core proteins are involved [16], [30], [31]. One such variant protein is τCstF-64 (gene symbol: *Cstf2t*), which is a paralog of the CstF-64 polyadenylation protein [18]. τCstF-64 is essential for spermatogenesis, as male *Cstf2t* knockout mice are infertile [21], [22], [23]. Most likely, τCstF-64 is necessary because it is involved in polyadenylation of genes critical for postmeiotic germ cell development. To examine the differences in mRNA species expressed in wild type and *Cstf2t^−/−^* mouse testes, we performed high-throughput sequencing of cDNAs using the 454 method (Fig. 1A) from 25-day postpartum (dpp) wild-type (∼65,000 reads) and *Cstf2t^−/−^* (∼90,000 reads) mouse testis RNAs (25 dpp was chosen because effects of *Cstf2t* are greatest at that age [21]). High-throughput cDNA sequencing was used because it offered an unbiased sampling of expressed genome sequences. Similar percentage of reads could be uniquely mapped to the mouse genome for each genotype (Fig. 1B). However, significant differences were found between wild type and *Cstf2t^−/−^* mice in the fraction of reads mapped to different regions of the genome (Fig. 1C). *Cstf2t^−/−^* mouse testis RNA showed dramatically decreased exonic regions and greatly increased intronic and intergenic regions represented in cDNAs. This indicated large-scale changes in transcriptional and mRNA processing patterns in these mice. Twelve percent of the reads from KO were mapped to genomic regions annotated as repetitive elements whereas only 5% of the reads from WT were so, consistent with our other results.

### Distal Polyadenylation Sites are used More Frequently in RNA From Testes of Cstf2t*^−/−^* Mice

Recent surveys have revealed that changes in patterns of poly(A) site usage from more distal sites to more proximal sites in proliferating cells such as cancer cells [26], [32], [33]. The relative usage of distal poly(A) site (RUD) score is a method for determining overall polyadenylation site use from microarray data [26], [27]. Examining both wild type and *Cstf2t^−/−^* samples, we note that RUD scores decrease progressively from 17 to 85 dpp (Fig. 2). This suggests that 3′ UTRs of testicular mRNAs shorten as these animals age. *Cstf2t^−/−^* mouse samples do not differ significantly from wild type samples at 17 dpp (Fig. 2) when appearance of τCstF-64-expressing pachytene spermatocytes is minimal [19]. However, at 22 dpp, when testis composition of pachytene spermatocytes increases [34], RUD scores of *Cstf2t^−/−^* mouse testis RNA are increased relative to wild type RNA (Fig. 2). The differences are more pronounced at 25 dpp and in adulthood (85 dpp), demonstrating that lack of τCstF-64 ameliorates partially the progressive decrease of 3′ UTR length seen in wild type mice. We note that differences in adult mice are also contributed to by changes in cell types expressed in the Cstf2t−/− mice [18]. This suggests that τCstF-64 is responsible for the progressive use of proximal polyadenylation sites uses in male germ cell development, and that in its absence more distal sites are used. A second hypothesis is that, in the absence of τCstF-64, transcription reads through distal polyadenylation sites and continues to intergenic regions. This latter hypothesis is supported by the finding that increased amounts of intergenic and intronic genomic regions in *Cstf2t^−/−^* mouse testis (Figure 1C). However, these hypotheses are not mutually exclusive, and both might be in effect.

### τCstF-64 Promotes the Expression of Intronless Small Genes (ISGs) in Mouse Testis

Further examination of microarray data from Dass *et al*. [21] suggested that mRNAs encoding intronless small genes (ISGs) were less abundant in *Cstf2t^−/−^* mouse testis (Fig. 3A). We confirmed these decreased levels using qRT-PCR for selected ISGs (*Cetn1, Hdhd1a, Papolb, Tssk1, Tssk2, Tssk6*) in testes from 25 dpp mice (Fig. 3B). However, mRNA levels of intron-containing genes *(Rps16, Actb)* did not decrease measurably in those same samples.

ISGs generally consist of expressed retroposons, cDNA copies of existing genes that are reinserted into the genome [35], [36]. ISGs are most prominently expressed in mammalian testis, most likely for spermatogenesis-specific functions and to compensate for MSCI [37], [38]. This leads us to propose the hypothesis that an important function of τCstF-64 is to control efficient polyadenylation of ISG mRNAs: *Cstf2t* is an expressed retroposon and thus an ISG [17], [18]. Because most testis-expressed ISGs – including τCstF-64– arose around 165 million years ago when mammals diverged from archosaurs [35], [39], germ cell-expressed ISGs must have co-evolved with τCstF-64. τCstF-64 therefore could accumulate specialized functions in polyadenylation while the more broadly expressed CstF-64 could maintain its more generalized functions. An additional possibility is that mRNA processing involving τCstF-64 would promote nuclear export of these non-intron containing transcripts [36].

### Polyadenylation Read-Through Increases for ISGs in *Cstf2t^−/−^* Mouse Testis

A leading hypothesis for how lack of τCstF-64 would affect polyadenylation and gene expression is that transcription will fail to terminate at typical polyadenylation sites, and instead will continue for up to several kilobases downstream [8], [40], [41]. Consequently, in *Cstf2t^−/−^* testes, we expected increased read-through of affected transcripts. We used quantitative reverse transcriptase-mediated PCR (qRT-PCR) to measure transcript abundance both upstream and downstream of reported sites of polyadenylation for two ISGs, *Cetn1* and *Tssk6* (Fig. 3B, C). These experiments were performed using both oligo(dT)-primed and random oligonucleotide-primed cDNAs with identical results We chose to show the random-primed results because they lack bias to potential changes in poly(A) addition. In Cstf2t−/− mouse testis RNA, we observed over 3-fold greater read-through in *Cetn1* gene transcription and nearly 1.7-fold read-through in *Tssk6* transcription than in wild type testis RNA, implying that 3′ end formation and transcription termination did not occur in the normal location for these genes. These data support a model in which τCstF-64 (and, by extension, CstF-64) is necessary for accurate placement of the 3′ end processing machinery at polyadenylation sites.

### LINE-1 Sequences Are More Abundant in *Cstf2t^−/−^* Mouse Testis RNA

We examined the sequences of intergenic (Fig. 4A), intronic (Fig. 4B), non-uniquely mapped (Fig. 4C) and unmapped (Fig. 4D) reads. These revealed significant overrepresentation of transposable elements in *Cstf2t^−/−^* mice, primarily LINE-1 sequences. Other repetitive DNA elements such as short interspersed nuclear elements (SINEs and LTRs) were also affected, although to a much lesser extent (Figs. 4A–D). These differences occur in both intergenic (Fig. 4A) and intronic regions (Fig. 4B). This suggests that LINE gene sequences are most highly represented in the intergenic and intronic regions that are affected by the *Cstf2t* knockout. There are two possible interpretations of this finding: first, LINE-1 sequence elements, including non-functional and fragmented sequences, are represented in these genomic regions and thus more highly expressed when those regions represented. The second interpretation is that loss of τCstF-64 somehow activates or de-represses LINE gene expression, perhaps by altering chromatin structure or by affecting LINE mRNA polyadenylation directly. This second interpretation is interesting, and we are designing future experiments to distinguish these hypotheses.

### Conclusions

Perhaps it was not surprising to see global changes in genome expression in the testes of *Cstf2t* knockout mice. Polyadenylation has long been linked to transcription and termination. Recent studies have even shown a role for both CstF-64 and τCstF-64 in histone mRNA expression in human epithelial cells [42], which might have further impact on euchromatin structure and expression. While those authors saw a strong effect of τCstF-64 on histone mRNA expression, we did not see evidence for a similar effect on germ cell histone variants in *Cstf2t^−/−^* mice (with our methodology, we would have detected only the polyadenylated variants [43]). Again, detection of effects on the class of ISGs should not have been surprising. As a class, these genes co-evolved with τCstF-64, and therefore might be assumed to have a full or partial requirement for it.

More surprising was the finding that LINE sequences but not other repetitive sequences such as SINEs were over-represented in *Cstf2t^−/−^* mouse testis. Two possible explanations come to mind: first, these are LINE fragments within intronic and intergenic sequences that are not full-length or active [44] and are therefore not physiologically relevant. More relevant, however is the second possibility that the absence of τCstF-64 has relieved repression of LINE mRNA expression in germ cells. An earlier report demonstrated LINE mRNA truncation by alternative polyadenylation in mouse fibroblasts [45]. This suggests the exciting possibility that τCstF-64 plays a role in control of LINE mRNA levels in germ cells. Future experiments will differentiate these possibilities.

## Supporting Information

Table S1 Primers used for quantitative RT-PCR.
(DOC)
