# Supplementary material for: The τCstF-64 Polyadenylation Protein Controls Genome Expression in Testis
Source: PLoS One. 2012 Oct 26;7(10):e48373. doi: 10.1371/journal.pone.0048373 (PMC3482194; doi:10.1371/journal.pone.0048373)
Supplement: Table S1 — Primers used for quantitative RT-PCR. (DOC) [file pone.0048373.s001.doc]

Table S1: Primers used for qRT-PCR

| **Gene** | **Primer 1** | **Primer 2** |
| --- | --- | --- |
| *Actb* | GGCTGTATTCCCCTCCATCG | CCAGTTGGTAACAATGCCATGT |
| *Cetn1* | TTGCCTCTACCAGCTACAAGA | CCGCTCCCATCAGAATCGAA |
| *Cetn1* DS | AGGCTTACATAACTCCAGGTCATC | GCAGAGCAGGCACTCTTTCA |
| *Gsg2* | CCACAGTGTGTGCAGCCAGT | GAGTCAGCATACAAAGGGTCG |
| *Hdhd1a* | TTGACCTGGACGGACTTATTCT | GGAACTCTACTATGGTCTGTGCG |
| *Papolb* | AAGCTGATCGAGACCCTCCA | CGGTAAGAGCCGAACGTAAAAA |
| *Rps16* | CACTGCAAACGGGGAAATGG | CACCAGCAAATCGCTCCTTG |
| *Rps16* DS | GTTTCTCTGTGTAGCCCTGAC | ACACCTTTTATCCCAGCACTC |
| *Tssk1* | CTCAAGCGACGAGGCTACATC | ACCGCCACGTTGAACTTTAGG |
| *Tssk2* | GCGGTCCTAAGGAAGAAGGG | TGACTGCCACATTGAACTTGAG |
| *Tssk6* | CGGGCGACAAACTCCTGAG | ACCGTCCCTTTATACTTCTTGGA |
